# Mental health profiles of migrants: a latent profile analysis of life satisfaction, psychological wellbeing, resilience and risk indicators

**DOI:** 10.3389/fpsyg.2025.1643759

**Published:** 2025-09-17

**Authors:** Fatma Coşkun, Semra Kiye

**Affiliations:** ^1^Department of Educational Measurement and Evaluation, Faculty of Education, Kahramanmaraş Sütçü Imam University, Kahramanmaraş, Turkey; ^2^Department of Guidance and Psychological Counseling, Faculty of Education, Muş Alparslan University, Muş, Turkey

**Keywords:** migrants, mental health, latent profile analysis, psychological wellbeing, depression, anxiety, stress, resilience

## Abstract

**Background:**

Migration is a global phenomenon that significantly impacts individuals' psychological wellbeing. Migrants often face a range of psychological stressors due to displacement, adjustment challenges, and trauma. Understanding how mental health indicators cluster in this population is essential for developing effective interventions.

**Aims:**

This study aimed to identify latent psychological profiles among migrants in Türkiye based on positive (life satisfaction, psychological wellbeing, resilience) and negative (depression, anxiety, stress) mental health indicators and to examine the demographic predictors of these profiles.

**Sample:**

The study included 436 adult migrants aged 18 to 64 residing in various provinces of Türkiye. A purposive sampling method was used to ensure diversity in characteristics such as gender, age, socioeconomic status, education level, and geographic location.

**Method:**

Participants completed validated self-report measures of life satisfaction, psychological wellbeing, resilience, depression, anxiety, and stress. Confirmatory factor analyses and reliability tests were conducted. Latent Profile Analysis (LPA) was used to identify distinct psychological profiles, and multinomial logistic regression was employed to examine demographic predictors of profile membership.

**Results:**

Four distinct psychological profiles were identified: (1) Moderate wellbeing and moderate risk, (2) high wellbeing and low risk, (3) high risk, and (4) low risk but moderate wellbeing. Age, gender, socioeconomic status, and education level were significant predictors of profile membership, while marital status was not. Higher age and lower income increased the likelihood of being in the high-risk group, whereas higher education was associated with better psychological outcomes.

**Conclusion:**

The findings highlight heterogeneity in migrants' psychological adjustment and underscore the importance of considering demographic diversity in mental health interventions. Tailored, culturally sensitive approaches may enhance the wellbeing and resilience of migrant populations.

## Introduction

Migration is an important phenomenon that has remained relevant from the past to the present, and its importance has been increasing in recent years. Migration is defined as a change of residence from one country to another that takes place over a meaningful period of time (Faist et al., 2013), and a clear understanding of migration processes provides insight into economic and social changes in social structures (Clark, 1986). Unlike in the past, today's social and political structures based on established modes of production have made migration a phenomenon that occurs in abnormal situations. Accordingly, individuals may migrate because they are forced to migrate because of their political or religious views or because of economic expectations (Dustmann and Glitz, 2011). In addition, migration may reflect the desire of individuals to improve their lives and the lives of their families, or it may be due to poverty, civil strife, extreme violence, war, human rights violations, persecution, and fear of being killed (Anisman et al., 2024). It is important for experts to better understand the impact of the migration phenomenon on both host communities and migrants, and to prepare intervention studies. In this regard, positive indicators such as life satisfaction (Raijman et al., 2022; Sánchez-Teruel and Robles-Bello, 2022), resilience (Cuervo et al., 2024; Qi et al., 2022), psychological wellbeing (Cebolla Boado and Suárez-Vergne, 2025), etc. as well as negative indicators such as depression (Wypych and Bilewicz, 2024), anxiety (Adzrago et al., 2024), and stress (Becerra et al., 2021) are being investigated, along with their prevalence, associated conditions, and causes.

Life satisfaction, as a separate structure and basic indicator (Pavot and Diener, 1993), including cognitive and universal assessment of quality of life, indicates how well the individual is developing physically and psychologically (Veenhoven, 1996). Research shows that high levels of psychological health, social support, and a sense of personal control increase life satisfaction among migrants (Lai and McDonald, 1995). Migrants have lower levels of life satisfaction in host countries with higher levels of binding social capital (inward-looking and homogeneous), while they have higher levels of life satisfaction in countries with higher per capita gross national income and lower rates of migration (Chu et al., 2018). Similarly, migrants' life satisfaction also increases with the sense of socio-economic success, the feeling of being less disadvantaged by being a foreigner, positive personal cost-benefit calculations regarding the decision to migrate, and loyalty to the host country (Raijman et al., 2022). When the changes in migrants' life satisfaction are analyzed by focusing on the characteristics of the host countries, it is seen that life satisfaction levels are higher in countries that offer a more hospitable social environment. In addition, migrants who adopt the strategy of integration in the public sphere for acculturation have higher levels of life satisfaction (Safak-Ayvazoglu and Kunuroglu, 2021). While acculturation does not have a significant effect on migrants' self-assessment of mental and physical health, it is understood that acculturation stress and resilience have indirect effects on migrants' physical and mental health (Serafica et al., 2019).

Resilience is basically defined as the ability to bounce back from difficult experiences and traumas and to cope with them in an adaptive way (Southwick and Charney, 2012) and is positively correlated with protective factors such as optimism, hope, self-efficacy and social support in migrants (Sánchez-Teruel and Robles-Bello, 2022). In the face of psychological distress in migrants, resilience manifests as a protective trait (Yu et al., 2014) and is positively associated with lower levels of psychological distress and higher levels of life satisfaction and happiness (Qi et al., 2022), but more exposure to trauma can lead to lower levels of resilience (Cuervo et al., 2024). Resilience can be achieved through various strategies in migrants. These strategies include cognitive reframing, behavioral adaptation, acceptance, sociability, courage, and/or cultural pride strategies, and it is believed that positive effects such as meaning, purpose, and hope occur in migrants' lives as a result of using these strategies (Garcini et al., 2022).

Like resilience, wellbeing is often associated with the availability of resources to cope with situations encountered in the host country and the use of effective coping strategies (Anisman et al., 2024). In addition, two ways to increase migrants' levels of adaptation are considered to be gaining knowledge about the new environment and increasing psychological wellbeing (Hu et al., 2022). As a vulnerable group, migrants may be more affected by social crises and more susceptible to crises. In such processes, the level of psychological wellbeing decreases for immigrants who face greater economic difficulties (Cebolla Boado and Suárez-Vergne, 2025). On the other hand, social isolation is also a risk factor for the psychological wellbeing of migrants. While social isolation is positively correlated with psychological distress and negatively correlated with life satisfaction and happiness, there is a significant relationship with psychological wellbeing and resilience plays a mediating role in this relationship (Qi et al., 2022). In a study conducted with elderly migrants, four different coping strategies were found to be used. These strategies were identified as low-resource, spouse-oriented, community-oriented, and multi-resource coping. In general, migrants with multi-resource coping strategies had the best psychological wellbeing. These qualities may enable them to mobilize their internal and external coping resources and draw on each to cope with adversity. Intrinsic strength and extrinsic support may also be mutually reinforcing, enabling these migrants to have a well-rounded coping strategy. Migrants in this group have the highest levels of education and health among the four groups. It has also been reported that community-oriented and multi-source coping strategies have significant buffering effects on psychological wellbeing among individuals with subjective health problems or low acculturation (Guo et al., 2022). Studies show that interventions aimed at increasing resilience and psychological wellbeing are effective in increasing migrants' adaptation by providing them with information about the new environment. These interventions also have positive effects on depressive symptoms (Hu et al., 2022).

Depressive symptoms are an important risk factor in migrants, and higher levels of depression and anxiety are found in migrants than in non-migrants in host countries, after controlling for age, gender, relationship status, highest level of education, employment, and health status (Demutska and Kiropoulos, 2021). In a study of African migrants in the United States, about 22 percent of participants reported moderate to severe symptoms of depression and anxiety (Saasa and Miller, 2021). In a study showing that knowing the language of the host country may have a positive effect on anxiety and depression levels, the situations of Anglo-Australian and Greek-born migrants born in Australia were examined. Greek-born immigrants who did not speak the language of the host country had higher levels of depression and anxiety and reported more depressive and anxiety symptoms than Anglo-Australian migrants (Kiropoulos et al., 2004). Latina migrant women were found to experience a range of stressors related to their migration and social position, and these factors were associated with increased symptoms of depression and anxiety. While 29% of these women reported moderate to severe depressive symptoms and 32% reported moderate to severe anxiety symptoms, stressors such as social isolation, perceived stress, and the stress of legal migration enforcement were associated with higher depression symptoms. Perceived stress and stress related to meeting basic needs were associated with higher anxiety symptoms, while social support received through positive social interactions was associated with lower anxiety symptoms (Ryan et al., 2021). Among Mexican adult migrants, normative stress was associated with anxiety after controlling for depression, while normative and acculturation stressors were associated with depression after controlling for anxiety. Acculturation and normative stress increased the likelihood of clinical illness, while social support reduced this risk and reduced depression by increasing resilience (Kiang et al., 2010). A meta-analysis of 17 studies of Arab migrants living in the United States, including 22,558 participants, showed an estimated prevalence of depression of 48 percent and anxiety of 58 percent, with alarming prevalence levels (El-Refaay et al., 2025).

Türkiye is one of the countries hosting a large number of migrants. Migrants in Türkiye are evaluated under the following categories: residence permit, irregular migration, international protection, and temporary protection. As of July 27, 2025, 1,107,387 migrants in Türkiye have a residence permit for 2025. In 2024, there were 225,831 irregular migrants and 9,009 people requesting international protection. Temporary protection status includes refugees, and the number of Syrian migrants with this status in 2024 was 2,901,478. Due to the civil unrest in Syria in 2011 and 2012, Türkiye adopted an open-door policy toward Syrian citizens. Consequently, a significant number of Syrian migrants have resided in Türkiye for over a decade. Analysis of the cities where Syrian migrants live shows that, although they tend to live in Istanbul for economic reasons, they prefer cities with dense Arab populations, such as Gaziantep, Sanliurfa, and Hatay (Republic of Türkiye Ministry of Interior, Directorate General of Migration Management, 2025). This may make it easier for them to adapt to life in Türkiye. However, despite certain religious and cultural similarities between Türkiye and Syria, differences in social norms, family structure, language, and other characteristics may make it difficult for migrants to adapt (Adigüzel and Tanyaş, 2020; Aslan and Güngör, 2019). In fact, migrants living in Türkiye cite gender roles, abuse, language barriers, and prejudice as significant obstacles to social cohesion (Karakurt and Gülerce, 2022).

As of September 16, 2020, there are seven temporary migrant accommodation centers in five provinces in Türkiye. These centers provide migrants with health services and places of worship, and they have established markets to meet their needs. Education services are also available for children, and adult education centers help adults without a profession gain one (Republic of Türkiye Ministry of Interior, Directorate General of Migration Management, 2025). These evaluations are important because they provide a better understanding of migrants living in Türkiye and the services provided to them. In particular, improving the limited knowledge of psychological data can inform interventions for migrants. In this context, research on the positive and negative mental health indicators of migrants living in Türkiye could contribute significantly to the literature on migration. For all these reasons, this study aims to examine the relationships between positive mental health indicators such as life satisfaction, resilience, psychological wellbeing and mental health indicators such as depression, anxiety, and stress among migrants living in Türkiye. In this context, the main research questions of this study are as follows:

*How many distinct latent psychological profiles exist among migrants in Türkiye based on positive and negative mental health variables?*

*Do the latent psychological profiles of migrants in Türkiye differ according to gender, age, marital status, socioeconomic status, and educational level?*

## Method

### Sample

This study was conducted with the participation of 436 migrants residing in Türkiye. The sample consisted of 436 individuals, 43.6% of whom were male and 56.4% female. Participants ranged in age from 18 to 64, with the most frequent age groups being 18 (7.8%), 21 (5.3%), and 40 (5.3%) years old. In terms of marital status, 56.2% of the participants were married, 41.1% were single, and 2.8% were divorced. Regarding socioeconomic status, the majority of the sample (58.9%) belonged to the low-income group with an income of up to 25,000 TL, followed by those with even lower income levels (27.1%) and the middle-income group (13.3%). In terms of education, the highest proportion of participants were university graduates (44.7%), followed by high school (15.8%) and middle school (14.0%) graduates. The participants held a variety of occupations, with the largest groups being students (16.7%) and housewives (~23%). A significant majority of the participants were originally from Syria (80.3%). With regard to their place of residence in Türkiye, the highest concentrations were in the provinces of Batman (12.6%), Gaziantep (11.7%), and Kahramanmaraş (10.6%). While the duration of residence in Türkiye varied, a substantial portion of the participants had been living in the country for over 10 years. In order to reflect the demographic diversity of migrants living in Türkiye, a purposive sampling method was used in this study. Participants were selected based on accessibility and willingness to participate, with attention given to diversity in terms of age, gender, country of origin, and province of residence. The sample was formed by purposefully selecting individuals who met the inclusion criteria relevant to the scope of the study. In this study, the sampling criteria were based on migrant individuals who have been residing in Türkiye with legal status for at least 1 year, are 18 years of age or older, have sufficient comprehension to complete the survey, and participated voluntarily.

### Data collection instruments

#### Satisfaction with life scale

As part of this study, the Satisfaction with Life Scale (SWLS), developed by Diener et al. (1985), was used to assess participants' life satisfaction. The scale was adapted into Turkish by Durak et al. (2010). The SWLS consists of five items that measure individuals‘ perceived quality of life. The items are rated on a 7-point Likert scale ranging from “1 = Strongly disagree” to “7 = Strongly agree.” There are no reverse-coded items in the scale. Higher scores on the SWLS indicate greater life satisfaction. The total score that participants can obtain from the scale ranges between 5 and 35. In this study, confirmatory factor analysis (CFA) indicated that the single-factor model provided a good fit to the data (χ^2^/*df* = 3.537, RMSEA = 0.76, GFI = 0.98, CFI = 0.98, NFI = 0.98, IFI = 0.98, SRMR = 0.02). Additionally, the Cronbach's alpha coefficient of the scale was found to be 0.81, indicating an acceptable level of internal consistency.

#### Psychological wellbeing scale

The Psychological wellbeing scale (P-WBS), developed by Diener et al. (2009) to assess individuals' psychological wellbeing, is a unidimensional instrument consisting of eight items. The scale was adapted into Turkish by Telef (2013). It employs a seven-point Likert-type format, with responses ranging from 1 = Strongly disagree to 7 = Strongly agree. All items are positively worded. Total scores range from 8 to 56, with higher scores indicating greater psychological wellbeing. In the present study, confirmatory factor analysis (CFA) supported the adequacy of the single-factor model, yielding acceptable fit indices (χ^2^/*df* = 2.68, RMSEA = 0.06, GFI = 0.97, CFI = 0.94, NFI = 0.95, IFI = 0.94, SRMR = 0.06). The internal consistency of the scale was high, as evidenced by a Cronbach's alpha coefficient of.93 (Diener et al., 2009; Telef, 2013).

#### The brief resilience scale

The Brief Resilience Scale (BRS), developed by Smith et al. (2008) to measure individuals' levels of psychological resilience, is a unidimensional instrument consisting of six items. The Turkish adaptation of the scale was conducted by Dogan (2015). It uses a five-point Likert-type response format, allowing participants to rate the items from 1 = Not at all appropriate to 5 = Completely appropriate. Three of the items are positively worded, while the remaining three are negatively worded. The total score ranges from 6 to 30, with higher scores indicating greater resilience. In the present study, confirmatory factor analysis demonstrated that the single-factor model provided an acceptable fit to the data (χ^2^/*df* = 6.131, RMSEA = 0.10, GFI = 0.98, CFI = 0.91, NFI = 0.90, IFI = 0.90, SRMR = 0.08). Additionally, the Cronbach's alpha coefficient for the scale was found to be 0.72, indicating an acceptable level of internal consistency.

#### Depression anxiety stress scale

The 21-item version of the Depression Anxiety Stress Scale (DASS-21) used in this study was adapted into Turkish by Yilmaz et al. (2017). The adaptation was based on the original version of the scale developed by Henry and Crawford (2005). The DASS-21 consists of three subscales—depression, anxiety, and stress—each measured by seven items. The scale uses a four-point Likert-type response format, with options ranging from 0 = Does not apply to me to 3 = Applies to me very much or most of the time. Scoring is performed separately for each subscale rather than using a total score. Thus, the possible score for each of the depression, anxiety, and stress subscales ranges from 0 to 21. In the current study, confirmatory factor analysis indicated that the single-factor model provided an acceptable fit to the data (χ^2^/*df* = 2.252, RMSEA = 0.054, GFI = 0.92, CFI = 0.95, NFI = 0.91, IFI = 0.95, SRMR = 0.04). Furthermore, the Cronbach's alpha coefficient for the total scale was found to be 0.94, indicating excellent internal consistency. The Cronbach's alpha values for the depression, stress, and anxiety subscales were 0.87, 0.87, and 0.86, respectively.

### Data analysis process

The aim of this study is to examine how many distinct latent psychological profiles exist among immigrant individuals in Turkey based on positive and negative mental health variables. To collect the data for the research, the “Satisfaction with Life Scale,” “Brief Resilience Scale,” “Psychological WellBeing Scale,” and “Depression Anxiety Stress Scale” were used. These scales, employed in the study, are widely recognized and well-established measurement tools in the literature.

Initially, confirmatory factor analysis (CFA) was conducted to test the structural validity of the scales in the sample used in this study. Subsequently, Cronbach's alpha reliability coefficients were calculated to determine the internal consistency of the scales. The primary fit indices used in the evaluation of CFA included chi-square/degree of freedom ratio (χ^2^/*df* ), RMSEA, GFI, CFI, NFI, IFI, and SRMR. Hooper et al. (2008) recommended the use of absolute fit indices, particularly for assessing the fit between a model and the sample data. In this context, they emphasized the importance of indices such as χ^2^, RMSEA, GFI, AGFI, RMR, and SRMR. Wheaton et al. (1977) indicated that the χ^2^/*df* ratio, developed to minimize errors due to sample size, should be below 5. RMSEA, an index evaluating how well the model fits the observed data, suggests acceptable fit with values below 0.08 (Hooper et al., 2008). GFI and AGFI values above 0.90 indicate a high level of fit between the model and the data (Hooper et al., 2008). SRMR, the standardized version of RMR, considers values below 0.05 as indicating excellent fit, and values below 0.08 as indicating adequate fit (Hu and Bentler, 1999). In contrast, comparative fit indices such as CFI and IFI indicate good model fit when their values exceed 0.90 (Bentler, 1995). In this study, these fit indices were used to assess the model's fit with the data.

After completing the validity and reliability analyses on the data obtained from the measurement tools used in the study, latent profile analysis (LPA) was performed to group participants based on their similar characteristics. This method aims to identify the number of latent groups in the data set. To determine the number of profiles that best represent the participants, several evaluation criteria were utilized. These criteria include the Bayesian Information Criterion (BIC), the number of model parameters (Npar), the proportion of each profile within the sample (cluster size), entropy value, and *p*-value as statistical significance tests. While BIC assesses the model fit, it also takes structural complexity into account; the number of parameters provides information about the model's flexibility. The entropy value indicates how clearly participants are assigned to the profiles, while the *p*-value tests whether the model is statistically significant. The combined examination of all these criteria allows for the determination of the optimal number of latent profiles for the data set (Asparouhov and Muthén, 2014).

In the final stage of the study, multiple logistic regression analysis was conducted to examine whether the identified latent psychological profiles significantly differed based on demographic variables (gender, age, marital status, socio-economic level, and education level). In this analysis, the latent profile group was defined as a multi-category dependent variable, and the demographic variables were included as independent variables in the model. The effect of each variable on the likelihood of profile membership was evaluated through parametric coefficients (B), odds ratios (Exp(B)), and significance levels (p). This method tested whether demographic characteristics significantly predicted the psychological profiles individuals belong to. Data preparation and regression analysis were conducted using SPSS 20.0, confirmatory factor analysis was performed with AMOS 24.0 software, and latent profile analysis was conducted using Latent Gold 6.0.

## Findings

Prior to conducting the analysis, descriptive statistics, including the mean, standard deviation, and correlation coefficients for the variables used in the latent profile analysis—life satisfaction, psychological resilience, psychological wellbeing, depression, anxiety, and stress—were computed (see Table 1). The results revealed that life satisfaction, psychological resilience, and psychological wellbeing were positively and significantly correlated with each other. In contrast, negative and significant relationships were found between these variables and depression, anxiety, and stress. Additionally, strong positive correlations were identified among the depression, anxiety, and stress variables (*r* = 0.614–0.731, *p* < 0.01). These robust correlations can be attributed to the fact that depression, anxiety, and stress are subscales of the same instrument, the DASS-21, and they reflect a shared structure of psychological distress. Following the descriptive statistics and correlation analyses, latent profile analysis (LPA) was conducted. This analysis was performed iteratively with different model solutions ranging from one to six profiles, and the fit indices for each model were compared. The detailed results of the model fit are presented in Table 2.

**Table 1:** Descriptive statistics and correlation coefficients for the variables used in the LPA.

**Variable**	**1**	**2**	**3**	**4**	**5**	**6**	**Mean**	**SD**
1. SWLS	1						19,09	8,014
2. P-WBS	0.323^**^	1					19,10	5,390
3. BRS	0.356^**^	0.402^**^	1				35,81	14,418
4. Depression	−0.190^**^	−0.345^**^	−0.409^**^	1			8,15	6,194
5. Anxiety	−0.292^**^	−0.387^**^	−0.465^**^	0.731^**^	1		8,11	6,151
6. Stress	−0.215^**^	−0.369^**^	−0.323^**^	0.614^**^	0.718^**^	1	9,18	6,006

Correlation is significant at the 0.01 level (2-tailed), SD, standard deviation.

**Table 2:** Comparison of model fit indices for different numbers of profiles (LPA Results).

**Model**	**BIC**	**Npar**	**Cluster size**	**Entropy *R*^2^**	**VLMR *P* value**	**Class.Err**.
1 Profile	17838,8783	12	(100%)	1,0000	-	0,0000
2 Profile	17128,6028	25	(59%) (41%)	0,8837	0,0000	0,0312
3 Profile	16867,1618	38	(59%) (30%) (11%)	0,9056	0,0000	0,0350
4 Profile	16719,2231	51	(48%) (26%) (15%) (11%)	0,8843	0,0000	0,0566
5 Profile	16667,4050	64	(46%) (26%) (15%) (7%) (6%)	0,8846	0,0130	0,0633
6 Profile	16658,2656	77	(29%) (26%) (23%) (9%) (7%) (6%)	0,8334	0,0001	0,1100

^**^Indicates statistical significance at the 0.01 level (*p* < 0.01). BIC, Bayesian information criterion; Npar, number of parameters; Entropy *R*^2^, classification accuracy; VLMR *p* value, Vuong–Lo–Mendell–Rubin test; Class. Err., classification error; Lower BIC and Class. Err., and higher entropy indicate better model fit.

According to the results of the latent profile analysis, a consistent decrease in the BIC values was observed as the number of profiles increased, indicating a gradual improvement in model fit. Notably, the models with two to four profiles demonstrated both a decline in BIC values and acceptably high entropy scores (>0.88), suggesting strong classification accuracy. The four-profile model showed a balanced structure with respect to both BIC (16719.2231) and entropy (0.8843), with a classification error rate of 5.66%. Although the five-profile model had the lowest BIC value, it did not show a meaningful increase in entropy and had a higher classification error rate (6.33%), which reduced the model's classification reliability. In the six-profile model, the entropy dropped to 0.8334, and the classification error increased to 11%, indicating a substantial decline in classification accuracy. Results of the VLMR test revealed that model improvement was statistically significant between two and four profiles (*p* < 0.001), but the improvement became less significant with the five-profile model (*p* = 0.0130). Taken together, these findings suggest that the four-profile solution provides the most optimal and parsimonious model in terms of both statistical validity and classification precision.

**Figure 1:**
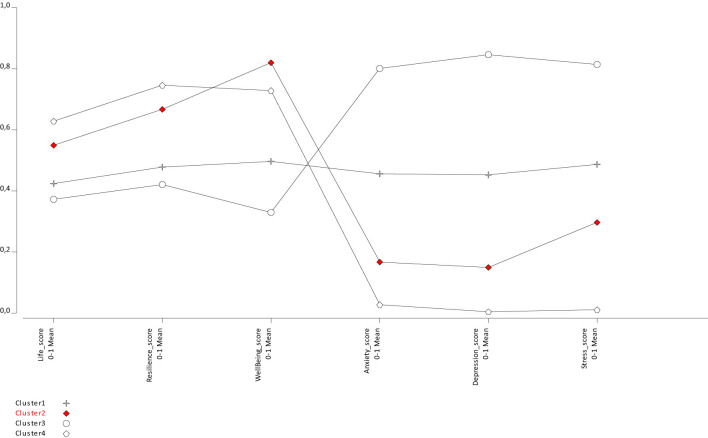
Profile-based mean values of mental health variables according to latent profile analysis.

Following the model comparisons presented in Table 2, the four-profile solution was identified as the most appropriate structure. Subsequently, the contents of these profiles were analyzed. Each profile was evaluated based on positive mental health indicators—such as life satisfaction, psychological resilience, and psychological wellbeing—and negative indicators—such as depression, anxiety, and stress. According to the results (Figure 1), the first profile was labeled as the “Moderate WellBeing and Moderate Risk Group” (Cluster 1). Individuals in this profile scored at moderate levels on life satisfaction (Life_score ≈ 0.48), resilience (Resilience_score ≈ 0.48), and psychological wellbeing (WellBeing_score ≈ 0.48). Similarly, their scores on anxiety (Anxiety_score ≈ 0.55), depression (Depression_score ≈ 0.55), and stress (Stress_score ≈ 0.48) also reflected a moderate risk level. This group consists of migrants who generally maintain a balanced, yet suboptimal level of psychological adjustment.

The second profile was labeled as the “High WellBeing and Low Risk Group” (Cluster 2). Individuals in this profile exhibited high mean scores on life satisfaction (Life_score ≈ 0.26), psychological resilience (Resilience_score ≈ 0.41), and psychological wellbeing (WellBeing_score ≈ 0.60). In contrast, they demonstrated low levels of anxiety (Anxiety_score ≈ 0.20), depression (Depression_score ≈ 0.20), and stress (Stress_score ≈ 0.36). This group consists of individuals with strong psychological resilience and high life satisfaction, coupled with low mental health risk.

The third profile was labeled as the “High Risk Group” (Cluster 3). This profile represents individuals with the highest level of psychological vulnerability. Their scores on life satisfaction (Life_score ≈ 0.15), psychological resilience (Resilience_score ≈ 0.14), and psychological wellbeing (WellBeing_score ≈ 0.14) were notably low. In contrast, their levels of anxiety (Anxiety_score ≈ 0.81), depression (Depression_score ≈ 0.81), and stress (Stress_score ≈ 0.74) were considerably high. This group consists of individuals who may be in urgent need of psychological support and intervention.

The fourth profile was labeled as the “Moderate WellBeing and Low Risk Group” (Cluster 4). Individuals in this profile reported moderate levels of life satisfaction (Life_score ≈ 0.55), psychological resilience (Resilience_score ≈ 0.50), and wellbeing (WellBeing_score ≈ 0.50), while also displaying very low levels of depression, anxiety, and stress (all ≈ 0.10). This suggests that although these individuals are not experiencing psychological distress, their positive mental health indicators remain moderate rather than high.

The second main objective of the study is to examine whether the latent psychological profiles of immigrant individuals in Türkiye differ significantly based on demographic variables such as gender, age, marital status, socio-economic level, and education level. In this context, a multiple logistic regression analysis was conducted in which the latent profile group was defined as the dependent variable, and the effects of each of these demographic variables on the profiles were tested (see Table 3). It was found that the model was generally significant (χ^2^(33) = 140.57, *p* < 0.001). The Nagelkerke *R*^2^ value was calculated as 30.3%, indicating that the model explained approximately one-third of the variance of the dependent variable. This suggests that the model has a moderate level of explanatory power. Additionally, the classification accuracy of the model was 55.7%, with a particularly high correct classification rate for the first profile (moderate wellbeing and moderate risk level) at 86.1%.

**Table 3:** Sociodemographic characteristics across latent psychological profiles (*n* = 436).

**Variable**	**Profile 1 *n* = 216 (49.5%)**	**Profile 2 *n* = 116 (26.6%)**	**Profile 3 *n* = 66 (15.1%)**	**Profile 4 *n* = 38 (8.7%)**	**χ^2^/*F***	***P***
Gender (%male)	43.5%	35.3%	48.5%	39.5%	11.08	0.011
Marital status (%single)	43.5%	37.2%	53.0%	42.1%	7.10	0.069
SES (%low)	64.5%	44.8%	71.2%	57.9%	25.76	<.001
Education (≥University)	44.4%	63.8%	33.3%	47.4%	71.33	<.001
Age (M ± SD)	35.8 (10.2)	32.7 (8.4)	41.6 (11.1)	36.2 (9.3)	20.42	<.001

χ^2^ tests were used for categorical variables; one-way ANOVA was used for age. M, mean; SD, standard deviation. *p* < 0.05 indicates significance.

When examined by variable, age emerged as a significant predictor in the model (χ^2^(3) = 20.42, *p* < 0.001). Specifically, age had a significant and positive effect when comparing the third profile (high-risk group) to the first profile (reference group) (B = 0.085, *p* < 0.001). This suggests that as age increases, individuals are more likely to belong to the high psychological risk group. Gender was also found to be significant (χ^2^(3) = 11.08, *p* = 0.011). Men were significantly more likely than women to be included in the second profile (high wellbeing, low risk) (B = 0.601, *p* = 0.017, OR = 1.82), suggesting that male immigrants may be psychologically more resilient or less at risk. Socio-economic status significantly explained profile differences (χ^2^(6) = 25.76, *p* < 0.001). Individuals with low income were much less likely to belong to the second profile (B = −1.549, *p* = 0.001, OR = 0.213), indicating that economic hardship may seriously impact the psychological wellbeing of immigrants. Education level was the strongest predictor in the model (χ^2^(18) = 71.33, *p* < 0.001). Specifically, individuals with middle school and high school education were significantly more likely to be included in the second profile (*p* < 0.05), with middle school graduates having an exceptionally high probability (B = 2.277, *p* = 0.002, OR = 9.75). The effects of other education levels were either limited or not significant. This suggests that attaining a certain level of education strengthens immigrants' psychological resources and increases the likelihood of being in lower-risk profiles.

The marital status variable is not statistically significant in the model (χ^2^(3) = 7.10, *p* = 0.069). Although being single was observed to increase the likelihood of being in the third profile (high-risk group), this finding is not statistically significant (*p* = 0.082). When these findings are considered overall, it can be concluded that the psychological profiles of immigrant individuals show significant differences based on demographic characteristics. Specifically, age, gender, education, and socio-economic status appear to be decisive factors in these differences, highlighting the importance of considering demographic diversity in mental health interventions.

## Discussion and conclusion

In this study, positive and negative mental health indicators related to the level of adaptation of migrants were investigated in the context of migrants living in Türkiye. The results show that migrants living in Türkiye form 4 different profiles in terms of positive and negative mental health indicators. These profiles are: (1) moderate wellbeing and moderate risk level, (2) high wellbeing and low risk level, (3) high risk level, (4) low risk but moderate wellbeing level.

Although those in the first profile have moderately positive mental health indicators, it is understood that they have the same level of risk. Being a migrant is a risk factor in itself. Having migrated at a young age is a statistically significant predictor of depressive symptoms (Abdul-Malak, 2020). This vulnerable group may be more affected by crises and may be more vulnerable to crises. For this reason, migrants may have low levels of psychological wellbeing (Cebolla Boado and Suárez-Vergne, 2025). The fact that most of the migrants in this study migrated due to the Syrian civil war, and likely lost some or all of their families, may explain the mental health picture of this group. The risk may be exacerbated by the large losses that migrants experience in the pursuit of wellbeing. In fact, there may be significant differences in mental health symptoms between migrants who left for attractive reasons, such as education and job opportunities, and those who left for pushing reasons, such as war and persecution. For instance, highly educated migrants with job prospects do not view economic inequality in their country of origin as an obstacle to life satisfaction (Kogan et al., 2018). Similarly, high levels of education and health, access to social resources such as work and school (Guo et al., 2022), and knowledge of the language of the migrated country (Kiropoulos et al., 2004) support migrant integration and ensure positive outcomes. On the other hand, although migrants show similar behaviors to natives when social crises occur, they are more vulnerable. The economic difficulties that arise in social crises, which can affect migrants to a greater extent, cause the psychological wellbeing of migrants to decline (Cebolla Boado and Suárez-Vergne, 2025). The fact that the socio-economic level of most of the migrants participating in the current study is quite low can be mentioned as another indicator of wellbeing levels. In a study conducted in Türkiye, it was found that refugees prefer to integrate in the public sphere in order to acculturate, but prefer to segregate in the private sphere. In addition, although they reported high levels of life satisfaction, they reported low levels of social wellbeing due to experiencing depressive emotions, re-experiencing old traumas through thoughts, and low levels of commitment and inclusion in the new context (Safak-Ayvazoglu and Kunuroglu, 2021).

Those in the second profile have high levels of positive mental health indicators and are at low risk. Considering that the migrants participating in this study were mostly from Syria, having fled and survived the civil war, as well as having migrated to a country (Türkiye) with culturally and religiously similar characteristics at certain levels, may have reduced the risk level by increasing the migrants' adaptation. In fact, when considering the effects of host country characteristics on changes in migrants' life satisfaction, it has been found that the effect of attachment to the host country on migrants' life satisfaction is strong (Raijman et al., 2022) and that migrants' life satisfaction is higher in countries that provide a more hospitable social environment (Kogan et al., 2018). However, despite many traumatic experiences, migrants remain strong by relying on both state support and positive beliefs about their spiritual beliefs (values) (Goodman et al., 2017). On the other hand, some migrants report that they feel well despite socioeconomic and cultural disadvantages. This is explained by their high level of integration into the mainstream culture, as well as their engagement with their background culture through distinctive experiences. Accordingly, when migrants' acceptance levels of the mainstream culture are high, their psychological wellbeing also increases with the use of social and individual resources (Güngör and Perdu, 2017). Additionally, the majority of the participants were Syrian and mostly resided in provinces with Arab populations, such as Batman, Kahramanmaraş, and Gaziantep, which may have facilitated their adaptation. However, it should be noted that, although Türkiye and Syria have certain similarities, they also have differences in characteristics such as social norms, family structure, and language. These differences may make it difficult for migrants to adapt (Adigüzel and Tanyaş, 2020; Aslan and Güngör, 2019).

The life satisfaction, resilience and psychological wellbeing levels of migrants in the third profile, which constitute the high-risk group, are quite low, while anxiety, depression and stress levels are quite high. It is known that positive indicators such as resilience are negatively correlated with anxiety and depression levels in migrants (Sánchez-Teruel and Robles-Bello, 2022). For example, as a very striking result, in a meta-analysis of 17 studies involving 22,558 participants of Arab migrants living in the United States, it was reported that the estimated prevalence of depression was 48% and the prevalence of anxiety was 58% (El-Refaay et al., 2025). The majority of the participants in the study live in provinces such as Kahramanmaraş, Batman and Gaziantep where the migrant population is very dense. The fact that these individuals experienced the earthquakes of 7.2 and 7.6 magnitude centered in Kahramanmaraş province of Türkiye on February 6, 2023 (Disaster and Emergency Management Authority (AFAD), 2025), which were named as the disaster of the century and in which more than 50 thousand individuals died (Disaster and Emergency Management Authority (AFAD), 2025), may make this picture more understandable. Indeed, this major disaster caused not only psychological but also socio-economic crises in Türkiye. In addition, the Covid-19 pandemic was experienced before this major disaster and many risk factors for migrants living in Türkiye (Mardin et al., 2020). Considering that the majority of the participants were Syrian migrants, the fact that migrants left their countries, families, lands and assets behind and were exposed to such a major natural disaster in their country of origin may have made them feel much more helpless. In fact, while being a migrant is associated with significant depressive symptoms (Yu et al., 2014), being exposed to more trauma reduces the level of resilience in migrants (Cuervo et al., 2024). On the other hand, migrants may be exposed to more victimization and difficulties, especially in times of crisis (Wypych and Bilewicz, 2024). In this context, it can be said that this group, which has been exposed to major social disasters such as the Covid-19 Pandemic since 2020 and the 6 February 2023 Kahramanmaraş earthquakes, may have been deeply affected.

The fourth profile includes those who score moderately on positive indicators of mental health and low on negative ones. When challenging situations arise, people try to cope with them in different ways. These methods can be either effective or ineffective. According to Baltaş (2000), ineffective methods such as generating negative thoughts, denial, suppression, and avoidance are also unhealthy. These individuals may use ineffective coping strategies, such as avoidance and suppression, instead of using effective coping mechanisms. Such coping mechanisms may limit improvements in to positive mental health indicators by preventing migrants from encountering different risks and acquiring diverse life experiences and social-external resources. Interacting with new people provides information about the new environment that enhances migrants' adaptation and makes interventions aimed at increasing their resilience and psychological wellbeing effective. Such interventions also have positive effects on depressive symptoms (Hu et al., 2022). However, migrants who are deprived of these interventions are not at high risk for negative indicators, but may maintain only moderate levels of wellbeing. For instance, avoiding work in the host country may spare them from the stress and anxiety of learning a new language. However, it may also prevent them from meeting new people and socializing. Furthermore, it may not lead to positive outcomes. Thus, they may miss opportunities to work and become financially self-sufficient. As a result, they may exhibit only moderate levels of positive mental health due to the absence of the psychological and material benefits of employment. Therefore, methods based on social isolation and avoidance can be considered as a risk factor on the psychological wellbeing of migrants. In fact, social isolation among migrants is positively related to psychological distress, negatively related to life satisfaction and happiness, and has a significant impact on psychological wellbeing (Qi et al., 2022). On the other hand, participants in this group may be using ineffective coping methods, such as denial, suppression, behavioral and mental letting go (Agargün et al., 2005). These methods may prevent them from accepting, facing, focusing on, and taking action regarding the current situation. For instance, an individual may be unable to accept being in a new country and cultural environment mentally or behaviorally. They may avoid their emotions by refusing to focus on them. While this may lead to lower levels of stress, anxiety, and depression, it may also result in only moderate levels of positive mental health indicators, as it creates obstacles to the individual's development. In this context, although this group did not use any active strategies to increase their adaptation levels, it may not contribute to the development of positive mental health indicators in the long term, even if it protects them from risks. According to research, increased resilience and wellbeing levels are often associated with immigrants using effective coping strategies to cope with problems encountered in the host country (Anisman et al., 2024), and the use of cognitive reframing, behavioral adaptation, acceptance, sociability, and courage strategies creates positive effects such as meaning, purpose, and hope in life (Garcini et al., 2022).

When demographic variables were examined, it was determined that while age, gender, socioeconomic level and education level were significant predictors in the model, marital status was not. Accordingly, it was determined that the probability of individuals being in the high psychological risk group increases as age increases. In support of this finding, it was determined that older migrants experienced higher acculturative stress than younger migrants (Yüksel and Fidan, 2021). On the other hand, the age of migration has a significant effect on the adaptation process. Accordingly, those who migrated at an older age exhibit lower adaptation levels than those who migrated at a younger age (Lee and Green, 2010; Steffen and Merrill, 2011). In addition, aging migrants cannot benefit sufficiently from social rights and especially the right to health (Yüksel and Hiçdurmaz, 2019). Research conducted in Türkiye shows that services provided to elderly migrants in the areas of socio-cultural adaptation, employment, socio-economic status, physical and mental health, and social support are limited (Apak and Artan, 2024). This data may explain why older migrants living in Türkiye are at risk. This age group may need more support socio-culturally and economically, and it may be more difficult to access support.

When gender is considered, male individuals are significantly more likely to be included in the second profile (high wellbeing and low risk) than females. For migrant individuals, being female, having poor health during childhood, and migrating at an early age are statistically significant predictors of depressive symptoms. Female migrants report higher rates of depressive symptoms compared to male migrants (Abdul-Malak, 2020). Women are more likely to experience anxiety/depression on a daily, weekly, or monthly basis compared to men (Adzrago et al., 2024). Latina migrant women have been found to experience a range of stressors related to their migration and social status, and these factors are associated with increased symptoms of depression and anxiety. While 29% of these women reported moderate to severe depressive symptoms and 32% reported moderate to severe anxiety symptoms, stressors such as social isolation, perceived stress, and migration enforcement stress have been associated with higher symptoms of depression (Ryan et al., 2021). The fact that the women who participated in this study were mostly housewives may have left them deprived of economic resources, status, and social supports. These deprivations may have placed women at higher risk, as social isolation is positively associated with psychological distress and negatively associated with life satisfaction and happiness in migrants (Qi et al., 2022). This result can also be considered a disadvantageous situation that arises from gender roles. In fact, gender roles, abuse, language barriers, and prejudice are identified as significant barriers to social integration for Syrian women seeking asylum whose husbands are missing. It has been shown that these women face high levels of risk and threats during the migration process (Karakurt and Gülerce, 2022).

The socio-economic level variable also explains the profile differences significantly. Individuals with low income levels are particularly unlikely to be in the second profile. This result shows that economic difficulties can seriously affect the psychological wellbeing of migrants. Accordingly, migrants have higher life satisfaction levels in countries with higher per capita gross national income and lower rates of migrants (Chu et al., 2018). While a strong sense of socio-economic success and positive personal cost-benefit calculations regarding the decision to migrate are associated with higher levels of life satisfaction (Raijman et al., 2022), economic difficulties that occur during social crises and can affect migrants to a greater extent reduce the psychological wellbeing of migrants (Cebolla Boado and Suárez-Vergne, 2025). On the other hand, acculturation stress and economic expectations in migrants are associated with higher levels of depression (Kiang et al., 2010). Migrants are less satisfied with their lives in host countries where economic inequality is higher (Kogan et al., 2018). On the other hand, although the migrants in this study have lived in Türkiye for over 10 years, 58.9% have a low income, and 27.1% have an even lower income. This situation can be explained by the cultural characteristics of Syrian migrants. Although the highest percentage of the group is university graduates (44.7%), the largest group is housewives (~23%). These findings may be related to the fact that Syrian women, despite their high level of education, are not considered suitable for employment, particularly in light of cultural characteristics. Indeed, although university graduates make up the largest group of participants, housewives make up the largest occupational group. These results appear to be consistent with the aforementioned cultural characteristics. Additionally, gender roles, abuse, language barriers, and prejudices are cited as significant obstacles for migrant women (Karakurt and Gülerce, 2022). Furthermore, a large proportion of participants hold “temporary protection” status. This status may limit their ability to transition to high-income professions and could negatively impact their socioeconomic status (Adigüzel and Tanyaş, 2020; Aslan and Güngör, 2019).

The education level variable stands out as the predictor with the strongest effect in the model. In particular, individuals who graduated from middle school and high school are significantly more likely to be included in the second profile. The effects of other education levels were found to be more limited or insignificant. This result shows that having a certain level of education strengthens the psychological resources of migrants and increases the likelihood of being included in low-risk profiles. In support of this finding, it has been determined that migrants with a higher level of education are more integrated than those with a lower level of education (Lee and Green, 2010). As migrants' education levels increase, their chances of employment and wages increase (Dudu, 2024). In addition, it was found that migrants with the multi-source coping strategy, which is the most effective among four different coping strategies in increasing the level of adaptation, have the highest level of education, health, and psychological wellbeing (Guo et al., 2022). On the other hand, highly educated migrants do not perceive the economic inequality of the host country as an obstacle to their level of life satisfaction (Kogan et al., 2018). An increase in the level of education positively affects the adaptation process by increasing the likelihood of migrants learning and using the language of the host country (Ataca and Berry, 2002; Yagmur and Van de Vijver, 2012). As the level of education of migrants living in Türkiye increases, their likelihood of learning and using Turkish increases, and this may have enabled them to socialize and integrate with society.

The marital status variable was not found to be significant in the model. Accordingly, being married or single is not distinguishing in terms of protective or risk factors. In support of the obtained result, it was found that whether migrants are married or single is not effective in terms of employment and income generation (Dudu, 2024). In addition, marital status has no effect on the perceived socio-economic status of migrants (Özer, 2024). However, unlike these results, being single among migrant women was found to be associated with lower levels of life satisfaction (Seker and Sirkeci, 2014). This difference may be related to the limited research conducted on migrants in Türkiye. It can be stated that it is important to increase the research conducted on migrants in Türkiye and to design them in a way that allows for more detailed results.

The majority of participants in this study are Syrian migrants under temporary protection. Following the civil unrest in Syria in 2011 and 2012, Türkiye adopted an open-door policy for Syrian citizens. Consequently, many Syrian migrants have lived in Türkiye for over a decade. As of September 16, 2020, 59,877 Syrians were under temporary protection in seven temporary accommodation centers across five provinces. Outside of these centers, an additional 3,559,041 Syrians under temporary protection live in Türkiye. Education services are available for all school-age children in the centers. Health services are provided at the same standard as for Turkish citizens. There are also places of worship and markets to meet the needs of foreigners. Adult education centers are also available, and those who do not have a profession are helped to obtain one (Republic of Türkiye Ministry of Interior, Directorate General of Migration Management, 2025). It can be concluded that these services will promote positive mental health among migrants and reduce negative indicators. However, the adaptation process and mental health symptoms require examining many dynamics, in addition to the support and services provided by the host country. It should also be noted that there may be many challenges associated with being a refugee (Adigüzel and Tanyaş, 2020; Aslan and Güngör, 2019; Karakurt and Gülerce, 2022).

## Limitations and future directions

This study has some limitations. The first of these is that the research data is based on scales that include individual reports from participants. Personal biases may have been involved in the scoring of the scales. In future studies, the research can be supported with qualitative data as well as quantitative data. These data can be obtained through interviews, observations, focus group discussions, etc. and the studies can be designed more robustly. The measurement tools used in this study are widely utilized across different cultures and supported by evidence of validity. At the outset, participants were required to have resided in Türkiye for at least 1 year to ensure a minimum level of cultural adaptation. During the data collection process, it was observed that the majority of participants had been living in Türkiye for over 10 years, suggesting a high level of cultural familiarity. Data were collected through face-to-face interviews, and translation support was provided when necessary to minimize potential misunderstandings due to language barriers. However, because the representation of specific migrant subgroups was limited in size, it was not possible to conduct cross-cultural measurement invariance analyses, which constitutes a limitation of the study. Furthermore, the purposive sampling strategy used in this research may restrict the generalizability of the findings, and this should be taken into account when interpreting the results.

Another limitation is the sample. The research sample consists largely of Syrian migrants. As is known, Syrian migrants living in Türkiye mostly settled in Türkiye as a result of very negative conditions during the Syrian civil war. Due to this situation, it can be stated that the results obtained can only be generalized to immigrants living in similar conditions. In addition, larger and more balanced samples can provide more generalizable results. Another limitation is that the current study did not collect data on the legal status of migrants, their reasons for migrating, or the social support they receive. This may have limited the discussion of the results. Future studies that collect such data can facilitate more comprehensive discussions. On the other hand, the cross-sectional nature of the data is another limitation. Designing future studies longitudinally and obtaining the data in this way can increase the reliability of the research results.

## Implications and recommendations

Today, the issue of migration should be addressed and understood in more detail. Indeed, migration is now a universal phenomenon. In countries such as Turkey (Turkish Migration Administration), which has a dense migrant population, it becomes necessary to make some regulations. In this respect, the results of the current research can form the basis for interventions to be carried out with migrants. In designing interventions suitable for individuals with different needs, preventive or curative interventions can be prepared by taking into account the different profiles obtained in this research.

The research results reveal the importance of developing positive mental health indicators in migrants. In this context, the development of resilience, life satisfaction, and psychological wellbeing capacities can be considered as strengthening components of the interventions to be prepared. In addition, as stated in the literature on the development of these capacities, it is thought that interventions that are both culturally sensitive and directly applicable will produce effective results in the adaptation of migrants. In this context, it can be suggested that the interventions to be prepared should also be designed in a multi-source and interactive manner, based on a culturally sensitive, systemic perspective. For instance, relevant Turkish institutions (e.g., Migration Management and the Ministry of National Education) could develop distinct psychoeducation programs for migrants to enhance their resilience, life satisfaction, and psychological wellbeing. These interventions can include both Turkish citizens and foreign nationals to promote socialization and participation. Similarly, these programs can be designed to reduce anxiety and stress symptoms or help individuals cope with them effectively. Mental health professionals working in temporary accommodation centers can be trained to lead these programs. Additionally, school counselors can implement these training programs in schools where Syrian children attend. To increase cohesion, the activities developed for these programs can incorporate cultural characteristics of both countries, such as food, important days, wedding and death ceremonies etc.

## Data Availability

The raw data supporting the conclusions of this article will be made available by the authors, without undue reservation.
